# Telemedicine-supported transition of stable coronary artery disease patients from tertiary to primary health care facilities: protocol for a randomized non-inferiority trial

**DOI:** 10.1186/s12913-016-1469-4

**Published:** 2016-07-07

**Authors:** Joanna d’Arc Lyra Batista, Mariana Vargas Furtado, Natan Katz, Milena Rodrigues Agostinho, Brasil Silva Neto, Erno Harzheim, Carisi Anne Polanczyk

**Affiliations:** Postgraduate Program in Epidemiology. Federal University of Rio Grande do Sul, Porto Alegre, Brazil; National Institute of Health Technology Assessment (IATS), Porto Alegre, Brazil; Hospital de Clínicas de Porto Alegre, Porto Alegre, Brazil; Postgraduate Program in Cardiology. Federal University of Rio Grande do Sul, Porto Alegre, Brazil; Instituto de Avaliação de Tecnologia em Saúde, Hospital de Clínicas de Porto Alegre - Centro de Pesquisas Clínicas, Rua Ramiro Barcelos, 2359 prédio 21 sala 507, Porto Alegre, RS CEP: 90035-903 Brazil

**Keywords:** Coronary artery disease, Telemedicine, Randomized clinical trial

## Abstract

**Background:**

Many Brazilian patients with complex diseases who are treated in tertiary referral clinics have been stable for long periods. The main needs of these patients involve monitoring of risk factors and review of drug prescriptions, which could be satisfactorily done in primary care facilities. The goal of this protocol is to evaluate the safety and effectiveness of telemedicine services to support the transition of patients with stable chronic coronary artery disease from the tertiary to the primary level of care.

**Methods/design:**

We designed a randomized non-inferiority protocol that will include 280 patients with stable coronary artery disease (for at least 12 months). Patients will be selected from the Ischemic Heart Disease Clinic in a tertiary care hospital in southern Brazil. Enrolled participants will be randomized into one of two groups: 12 months of follow-up at the same clinic; or 12 months of follow-up at a primary care facility with clinical support from a telemedicine platform including a toll-free line for physicians (intervention group). In the intervention group, decisions to refer patients to tertiary care during follow-up will be made jointly by primary physicians and medical teleconsultants. The groups will be compared in terms of the primary outcome—maintenance of baseline functional class 1 or 2 after 12 months. Secondary outcomes include control of risk factors and instability of the disease.

**Discussion:**

We intend to determine the effectiveness of using telemedicine to qualify the transition of patients with chronic coronary disease from the tertiary to the primary level of care. This should facilitate the access of patients to the healthcare system, since care will be provided closer to their homes, and provide more opportunities for treatment of severe cases at tertiary care hospitals that are often overcrowded.

**Trial registration:**

ClinicalTrials.gov # NCT02489565 – trial registration date May 13, 2015

## Background

Coronary artery disease (CAD) is characterized by a wide range of clinical presentations. In many patients, CAD remains stable for long periods, with fairly steady symptoms over time, whereas in others the presence of multiple cardiovascular events entails the need for revascularization [1]. With a greater number of diagnoses and increased survival following treatment of acute ischemic CAD, the number of patients experiencing stable angina has increased progressively worldwide. In Brazil, 18 thousand new cases of CAD and 900 thousand new cases of angina pectoris are expected every year [2].

The Brazilian public health care system—the Unified Health System (SUS)—was designed to provide universal, comprehensive healthcare to all Brazilian citizens. However, complex technologies, financial resources, and qualified personnel are currently concentrated in large cities, with marked regional inequality [3]. In Porto Alegre, state capital of Rio Grande do Sul, only about 50 % of CAD patients treated in a specialized outpatient clinic at a tertiary care university hospital (Hospital de Clinicas de Porto Alegre, HCPA) live in the city. The other 50 % are referred from other cities in the state Rio Grande do Sul. Some patients travel as much as 8 h to reach the hospital. This situation also causes overcrowding of the tertiary clinic and restricts access, making it difficult to book urgent appointments and prioritize the most severe cases. The performance of specialized tests and exams is often delayed.

Nevertheless, many of the patients seeking care in Porto Alegre could be managed in their city of origin within the context of primary care, defined as “that aspect of a health services system that assures (…) comprehensiveness of care in the sense that only rare or unusual manifestations of ill health are referred elsewhere, and coordination of care such that all facets of care (wherever received) are integrated” [4]. SUS is based on a logic of referral and counter-referral, according to which less technologically dense services may refer patients for care at greater complexity facilities, namely, hospitals and specialized clinics; and these complex services can refer the patient back for follow-up at primary care facilities that are closer to the patient’s home [5, 6]. Because of structural limitations, including health care professionals who feel unprepared or unsupported to treat situations they deem as complex, the referral and counter-referral system is often bypassed.

In this context, telemedicine provides a useful tool for diagnostic and therapeutic support as well as continuing health education. Telehealth appointments involving workers, health professionals, and managers can be used to clarify doubts about clinical procedures and health actions and answer questions regarding the work process [7].

This article describes a protocol designed to evaluate the use of telemedicine to support the counter-referral of stable CAD patients treated in a tertiary outpatient clinic to primary care facilities, with remote assistance for routine follow-up and decision-making regarding the need for care at the tertiary clinic in the presence of clinical instability. This initiative is intended to facilitate the access to tertiary services for patients with more severe conditions, reduce delays in booking appointments and tests, and ensure delivery of quality care to counter-referred patients.

## Methods/design

### Study design

This randomized non-inferiority trial was designed to compare two groups: CAD patients meeting outpatient clinic discharge criteria, counter-referred from tertiary to primary care for follow-up with telemedicine monitoring (intervention group); and CAD patients meeting outpatient clinic discharge criteria with follow-up at the original tertiary care outpatient clinic (control group).

### Study population and sample size

The study population will be selected among patients from the Ischemic Heart Disease outpatient clinic at HCPA, a tertiary care university hospital in South Brazil. All clinic patients have CAD defined by the presence of at least one of the following: documented history of myocardial infarction, surgical or percutaneous myocardial revascularization, lesion > 50 % in at least one coronary artery assessed by angiography, or presence of angina and positive noninvasive testing of ischemia [8].

Study participants will undergo standard anamnesis and medical examination to verify clinical stability and discharge criteria.

The sample size was calculated as 140 patients in each group—considering a non-inferiority margin of 10 % and incidence of 92 % of the main outcome, that is, maintenance of Canadian Cardiovascular Society Functional Classification (CCS) 12 months after the start of follow-up. This was based on the rate of patients with increase in angina to a higher CCS reported by a previous study for the control group receiving optimal treatment for CAD [9]. An additional 10 % margin was added to account for losses to follow-up. For calculation of sample size, a ß of 0.9 and an α of 0.05 (two-sided) were considered.

### Inclusion and exclusion criteria

Individuals of both genders, aged ≥ 18 years, with a diagnosis of CAD and CCS I or II at the moment of enrollment, without cardiovascular events requiring hospital admission during the previous year, will be selected for the study. Verification of discharge criteria will be performed by the medical team running the clinic, without interference from the investigators.

Patients who have been followed-up at the clinic for less than 1 year, unstable patients needing medication adjustments, patients performing diagnostic evaluation at the moment of enrollment, or those who do not agree with the outcome of randomization will be excluded.

### Interview and questionnaires

**Fig. 1:**
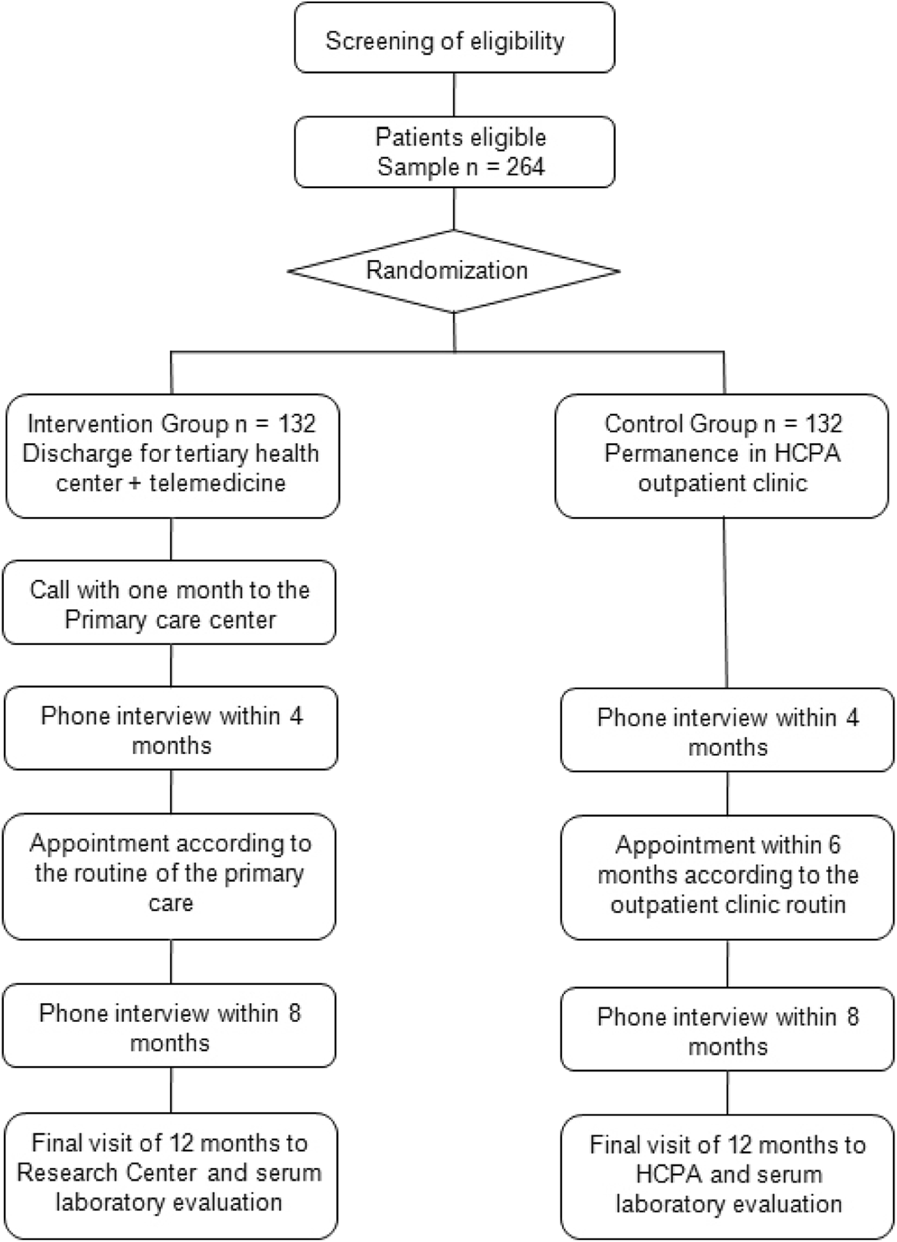
Flow Diagram of Study Design

Eligible individuals will be invited at the outpatient clinic, after their regular appointment, to participate in the study. At study entry, patients will undergo a baseline interview (Fig. 1). Interviews will be performed by a trained professional using a standard pre-coded questionnaire. The following information will be collected by interview or review of medical records: (i) clinical and epidemiological data: previous diagnoses, including chronic heart failure, peripheral arterial disease, acute myocardial infarction (AMI), chronic arterial disease, chronic obstructive pulmonary disease (COPD), dyslipidemia and diabetes; Canadian Cardiovascular Society (CCS) Functional Classification of Angina grade at the moment of the interview [10]. (ii) Biological factors: age and sex. (iii) Socioeconomic factors: skin color, social class, literacy, income, and schooling. (iv) Lifestyle: alcohol consumption, smoking, and practice of physical activities. (v) Use of medications: dose and type of medications, adherence to drug treatment (measured by an adapted version of the Brief Medication Questionnaire). (vi) Laboratory tests and medical procedures performed.

### Intervention

The intervention will be discharge from the tertiary outpatient clinic and referral to the primary care facility nearest to the home address, with clinical support from an existing telemedicine platform and a toll-free hotline (TelessaúdeRS/UFRGS). Prompt access to the tertiary outpatient clinic will be ensured in acute situations following agreement between primary physicians and teleconsultants. The toll free line is accessible to primary care physicians is available Monday through Friday, from 8:00 a.m. to 5:30 p.m. [11].

A TelessaúdeRS/UFRGS team designated for the project includes one specialized cardiologist with access to the patient’s electronic record, whose role will be to aid primary care physicians in patient management, and a medical student dedicated to manage and monitor the study flowchart.

The control group will undergo the routine follow-up scheme, with appointments scheduled every six months or once yearly at the outpatient Ischemic Cardiopathy Clinic at HCPA.

### Randomization

Patients eligible for inclusion will be randomized 1:1 using random block sizes of six and four, by a computerized list provided by randomization.com. The investigator in charge of randomization will not be involved in determination of eligibility or assessment of outcomes. Group assignment will be performed by opening a sealed opaque envelope in front of the patient after signature of the informed consent form and completion of the initial research questionnaire. After that, the groups will be managed as follows (Fig. 1):

- Telehealth group (intervention group): the patient will be discharged from the tertiary care outpatient clinic and directed to a primary care facility (Basic Health/Family Health Strategy unit) previously identified according to residential address. At the primary care facility, patients will book a routine appointment within two months. For that, the patient will bring the discharge note, comprising all relevant information about the case, medical prescriptions, and a counter-reference note with therapeutic planning. The TelessaúdeRS/UFRGS team will contact the basic health unit and provide access to the telehealth platform for follow-up monitoring. A teleadvisory will be scheduled between the medical teleconsultants and the primary physicians within two months after outpatient clinic discharge to discuss clinical management of the patient. Furthermore, primary physicians can call the support team at TelessaúdeRS/UFRGS whenever they have doubts about the clinical management. Patients will be interviewed by telephone at four and eight months of follow-up using the standardized instrument used at study entry. The calls will be made by independent evaluators, hired and trained specifically for this purpose. An appointment will be booked at 12 months of follow-up for final evaluation of the patient.
- Control group: patients will undergo the usual flow routine during the study period. Patients will be interviewed by telephone using the standardized instrument at four and eight months of follow-up. An appointment will be booked at 12 months of follow-up for final evaluation of the patient. Ifclinical status is stable, the patient will no longer attend the tertiary outpatient clinic for follow-up.

For all patients (intervention and control groups), the 12-month appointment will focus on evaluation of symptoms, episodes of disease decompensation, and adherence to treatment. Patients who do not show up for the 12-month appointment will be contacted by telephone. If the patient is not found, the study team will contact the primary care facility to determine the patient’s clinical status.

### Outcome measures and assessment procedures

Telephone interviews aim to identify changes in functional class or clinical events taking place over the follow-up period.

The final 12-month interview will evaluate the same components assessed in the initial interview, in order to determine improvement/worsening of clinical status. All patients will undergo blood tests with serum lipids and glucose evaluation.

The primary outcome is maintenance of CCS I or II at 12 months.

Secondary outcomes focus on risk factors. Control of risk factors will analyze variations of at least 5 % in the following pre and post-intervention measurements [12]:

- systemic blood pressure;
- controlled dyslipidemia: LDL < 100 mg/dL;
- glycated hemoglobin (in the presence of diabetes);
- abstinence from tobacco smoking;
- about 150 min/week of regular physical activity.

Additional secondary outcomes include health care access variables, such as: medical appointment in primary care facility taking place within not more than six months after referral, with review of medications and issue of prescriptions; and disease instability during the 12-month follow-up, defined as the need for seeking emergence care due to cardiovascular disease: acute chest pain; heart failure decompensation; acute arrhythmias; stroke.

Given the objective nature of the outcomes, the safety of the intervention, and the brief follow-up period, endpoint adjudication and data monitoring committees will not be appointed. No interim analyses are proposed. At the 4 and 8-month telephone interviews patients will be asked about possible unintended effects of the trial, and will receive guidance as required. After the trial, patients will be readmitted to the outpatient clinic if continued care at a primary care facility is not available.

### Statistical analysis

Continuous variables without normal distribution will be described as mean ± standard deviation or median and inter-quartile range (IQR). Categorical variables will be expressed as percentages. Comparisons between groups will be performed by Student’s *t*-test for continuous variables with normal distribution and Wilcoxon’s test for variables without normal distribution. Fisher’s exact test will be used for categorical variables. Logistic regression analysis will be used to establish the effect of the counter-referral strategy on primary and secondary outcomes at 12 months. Analysis of survival free of events at 12 months will be performed using the Cox multivariate regression and described in Kaplan-Meier survival curves. Variables with *P <* 0.10 in the univariate analyses will be included in the multivariate model. Primary outcome analysis will be according to intention to treat (ITT). Determination of sample size has taken into consideration a 10 % loss to follow-up.

Data will be analyzed in SPSS 18.0 for Windows. Statistical significance is set at *P <* 0.05.

### Ethical precautions

The study will be carried out in accordance with the guidelines for Good Clinical Practice, the Declaration of Helsinki, the Medical Research Involving Human Subjects Act (WMA) and other applicable guidelines, regulations and acts. Ethical approval was obtained from the Institutional Review Board at Hospital de Clinicas de Porto Alegre (CAAE: 28064614.0.0000.5327). All participants will be extensively informed about the study, including confidentiality aspects and the right to withdraw from the study at any time, without providing an explanation and with no effect on the quality of treatment. Written information will be provided prior to inclusion and patients will express their agreement to participate by signing a written consent form. The name and telephone number of a physician will be provided for patients with questions about the research before, during, and after the start of the protocol.

Study sponsor and funding agencies have not participated in study design and will not be involved in collection, management, analysis, and interpretation of data; writing of the report; and the decision to submit the report for publication. Because this is a single-center study, a steering committee was not appointed.

Results will be submitted for publication in a peer-reviewed journal after the end of the trial, without restrictions. Authorship will be defined according to the recommendations of the International Committee of Medical Journal Editors.

## Discussion

Tertiary care services in Brazil are overcrowded, with reduced capacity to accept new patients and schedule new appointments. At the HCPA Ischemic Cardiopathy Clinic, in the city of Porto Alegre, more than 50 % patients come from other cities, located as far as 8 h away. These patients face stressful trips and risk of road accidents, among others. Only two new patients are evaluated at the clinic every week. The large patient volume also compromises the ability to provide urgent appointments, prioritize more severe cases, and perform test such as echocardiograms and myocardial scintigraphy. However, many of the patients seen at the clinic have been stable for long periods, requiring only monitoring of risk factors and re-evaluation of prescribed medicines. These activities can be satisfactorily performed at a Basic Health Unit/Family Health Program, with prompt referral to the tertiary hospital only in the presence of instability.

It is believed that the mechanisms facilitating patient referral and counter-referral are fundamental to assure the principle of comprehensiveness, suitability of care for the patient, and rational use of resources [13]. Telehealth centers can help improve the effectiveness of counter-referral and management of stable chronic cases, thus preventing the overcrowding of specialized services and ensuring good quality follow-up near the patient’s home [14]. A previous protocol carried out by our telehealth program (TelessaúdeRS/UFRGS) showed that referral to a higher level of care was avoided in one out of every two cases following discussion with telehealth specialists [15].

The main risk for participants of the present protocol is violation of data confidentiality. All possible measures will be taken by investigators to prevent this. A single patient identification number will be used. Direct benefits are not expected either. However, the information obtained will serve to improve the management of patients with stable CAD. The results of this work will also provide important information on the best way to allocate technological resources in this area.

It is expected that the quality of follow-up and monitoring of ischemic cardiopathy will be similar in the tertiary clinic and with the primary care/telemedicine scheme. If that is confirmed, stable patients will in the future have access to treatment close to their homes, while opening space for more severe patients who have no choice but to be treated in a tertiary hospital.

## Abbreviations

SUS, Unified Health System from Brazil; HCPA, Hospital de Clinicas de Porto Alegre; CAD, Coronary Artery Disease; AMI, Acute Myocardial Infarction; COPD, chronic obstructive pulmonary disease; CCS, Canadian Cardiovascular Society; TelessaúdeRS, Telehealth center from Rio Grande do Sul; UFRGS, Federal University of Rio Grande do Sul; LDL, Low Density Lipoprotein; IQR, inter-quartile range; WMA, World Medical Association; CAAE, Certificate of Presentation for Ethical Consideration

## References

[1] Weil BR and Canty Jr JM. Coronary Blood Flow and Myocardial Ischemia. Essential Cardiology. 2013;doi:10.1007/978%E2%80%931%E2%80%934614%E2%80%936705%E2%80%932_22.

[2] Bueno FR, Correa FR, Alves MAS, Bardin MG, Modesto JA, Dourado VZ (2012). Physical exercise capacity and its prognostic value in postoperative cardiac surgery [Portuguese]. Fisioter Mov.

[3] Mattos LAIP, Berwanger O, Santos ES, Reis HJL, Romano ER, Petriz JLF (2013). Desfechos clínicos aos 30 dias do registro brasileiro das síndromes coronárias agudas (ACCEPT). Arq Bras Cardiol.

[4] Starfield B (2011). Basic concepts in population health and health care. J Epidemiol Community Health..

[5] Starfield B (2012). Primary care: an increasingly important contributor to effectiveness, equity, and efficiency of health services SESPAS report. Gac Sanit.

[6] Fihn SD, Gardin JM, Abrams J, Berra K, Blankenship JC, Dallas AP (2012). ACCF/AHA/ACP/AATS/PCNA/SCAI/STS Guideline for the diagnosis and management of patients with stable ischemic heart disease: a report of the American College of Cardiology Foundation/American Heart Association Task Force on Practice Guidelines, and the American College of Physicians, American Association for Thoracic Surgery, Preventive Cardiovascular Nurses Association, Society for Cardiovascular Angiography and Interventions, and Society of Thoracic Surgeons. Circulation.

[7] Brazil. Ministry of Health. Portaria n. 2546, 2011. http://bvsms.saude.gov.br/bvs/saudelegis/gm/2011/prt2546_27_10_2011.html. Accessed 25 June 2015.

[8] Cesar LA, Ferreira JF, Armaganijan D, Gowdak LH, Mansur AP, Bodanese L (2014). Guideline for Stable Coronary Artery Disease. Arq Bras Cardiol.

[9] Wilson SR, Scirica BM, Braunwald E, Murphy SA, Karwatowska-Prokopczuk E, Buros JL (2009). Efficacy of ranolazine in patients with chronic angina observations from the randomized, double-blind, placebo-controlled MERLIN-TIMI (Metabolic Efficiency With Ranolazine for Less Ischemia in Non-ST-Segment Elevation Acute Coronary Syndromes) 36 Trial. J Am Coll Cardiol.

[10] Canadian Cardiovascular Society Functional Classification of Angina. Cardiovascular Disability: Updating the Social Security Listings. 2010. http://www.ncbi.nlm.nih.gov/books/NBK209964/. Accessed 20 July 2015.

[11] Damasceno F, Reategui E, Schmitz CAA, Harzheim E, Epstein D (2014). Supporting Teleconsulting with Text Mining: Continuing Professional Development in the TelehealthRS Project In: Collaboration and Technology.

[12] U.S. Department of Health and Human Services, National Institutes of Health. NIH Publication n. 05–3290. 2005. http://www.nhlbi.nih.gov/health/resources/heart/heart-cholesterol-hbc-what-html. Accessed 13 July 2015

[13] Fratini JG, Saupe R, Massaroli A (2008). Referência e contra referência: contribuição para a integralidade em saúde. Cienc Cuid Saude.

[14] Davis DA, Thomson MA, Oxman AD, Haynes RB (1995). Changing physician performance. A systematic review of the effect of continuing medical education strategies. JAMA.

[15] Gusso G, Lopes JMC (2012). Tratado de Medicina de Família e Comunidade – 2 Volumes: Princípios, Formação e Prática.

